# Zidebactam restores sulbactam susceptibility against carbapenem-resistant *Acinetobacter baumannii* isolates

**DOI:** 10.3389/fcimb.2022.918868

**Published:** 2022-07-08

**Authors:** Jose Cedano, Michelle Baez, Fernando Pasteran, Sabrina Daiana Montaña, Grace Ra, Venjaminne Fua, Alejandra Corso, Marcelo E. Tolmasky, Robert A. Bonomo, María Soledad Ramírez

**Affiliations:** ^1^Center for Applied Biotechnology Studies, Department of Biological Science, College of Natural Sciences and Mathematics, California State University Fullerton, Fullerton, CA, United States; ^2^National Regional Reference Laboratory for Antimicrobial Resistance (NRL), Servicio Antimicrobianos, Instituto Nacional de Enfermedades Infecciosas, Administracion Nacional de Laboratorios e Institutos de Salud (ANLIS) “Dr. Carlos G. Malbrán”, Buenos Aires, Argentina; ^3^Laboratorio de Bacteriología Clínica, Departamento de Bioquímica Clínica, Hospital de Clínicas José de San Martín, Facultad de Farmacia y Bioquímica, Buenos Aires, Argentina; ^4^Research Service and GRECC, Louis Stokes Cleveland Department of Veterans Affairs Medical Center, Cleveland, OH, United States; ^5^Departments of Medicine, Pharmacology, Molecular Biology and Microbiology, Biochemistry, Proteomics and Bioinformatics, Case Western Reserve University School of Medicine, Cleveland, OH, United States; ^6^CWRU-Cleveland VAMC Center for Antimicrobial Resistance and Epidemiology (Case VA CARES), Cleveland, OH, United States

**Keywords:** Acinetobacter, carbapenem-resistance, zidebactam, sulbactam, DBOs, synergy, susceptibility

## Abstract

Carbapenems are commonly used to treat infections caused by multidrug-resistant (MDR) bacteria. Unfortunately, carbapenem resistance is increasingly reported in many gram-negative bacteria, especially *Acinetobacter baumannii*. Diazabicyclooctane (DBO) β-lactamase inhibitors, such as avibactam (AVI), when combined with sulbactam successfully restore sulbactam susceptibility against certain carbapenem-resistant *A. baumannii* (CRAB) isolates. In the present study, we tested zidebactam, a novel DBO with an additional mechanism of action, in combination with sulbactam against CRAB isolates, including strains that exhibited resistance against sulbactam/avibactam combination. A panel of 43 geographically and genetically distinct CRAB isolates recovered from different hospitals and containing different mechanisms of resistance were included in the present study. We also tested three reference strains (AB0057, AB5075, and AYE). Minimum inhibitory concentrations (MICs) for sulbactam (range 0.12–512 mg/l) and sulbactam plus 4 mg/l zidebactam were performed using microdilution according to CLSI Standards. A decrease ≥2 dilutions in sulbactam MICs was observed in 84% of the isolates when tested in combination with zidebactam. The sulbactam/zidebactam combination was able to restore sulbactam susceptibility in 91% of the isolates, including isolates that were resistant to sulbactam/avibactam combination. These data encouraged us to further explore sulbactam/zidebactam in other experimental models especially against CRAB isolates resistant to other DBOs.

## Introduction

*Acinetobacter baumannii* is a Gram-negative nosocomial bacterium often found to be multidrug resistant (MDR) that can cause pneumonia, bacteremia, and wound infections associated with high mortality rates (Spellberg and Bonomo, 2014; Garnacho-Montero and Timsit, 2019; Karakonstantis et al., 2020). *A. baumannii* resistance to carbapenems (CRAB) is frequently reported in hospital settings (Piperaki et al., 2019; Ramirez et al., 2020). The World Health Organization (WHO) has designated *A. baumannii* as a “high-priority pathogen” for the research and development of antibiotics, and in 2019, the CDC reported it as an “Antibiotic Resistance Threat” due to its non-susceptibility to carbapenems. Carbapenems are usually prescribed for high-risk and difficult-to-treat bacterial infections.

To meet the challenge of difficult-to-treat infections, a conventional approach was undertaken to develop β-lactamase inhibitors (van Duin and Bonomo, 2016). The diazabicyclooctane (DBO) β-lactamase inhibitors have been paired with cephalosporins and carbapenems to restore antibiotic efficacy and preserve partner susceptibility (Barnes et al., 2019; Tooke et al., 2019). However, against multidrug-resistant (MDR) *A. baumannii* isolates the efficacy of the current clinical combinations is uncertain. Novel experimental combinations such as sulbactam/avibactam demonstrated promising activity against CRAB with different genetic backgrounds; however, the combination was ineffective against MBL-expressing CRAB (Rodriguez et al., 2020; Pasteran et al., 2021). Sulbactam/durlobactam (ETX2514) is also a promising combination in development that has shown very favorable activity against CRAB isolates, except in MBL producers (Barnes et al., 2019).

Zidebactam is a novel β-lactam enhancer with high affinity and specific binding to PBP2 of all the clinically relevant Gram negatives including *Pseudomonas aeruginosa* and *A. baumannii* and therefore possesses intrinsic antibacterial activity against a large majority of Enterobacterales and *Pseudomonas aeruginosa* (Moya et al., 2017; Sader et al., 2017) and acts as a β-lactam enhancer in combination with PBP3-binding partner β-lactam. Zidebactam also inhibits a wide variety of β-lactamase enzymes such as Ambler class A, and C; however, it is not an inhibitor of Ambler class D β-lactamase such as *Acinetobacter*-associated OXA-carbapenemases (Papp-Wallace et al., 2018). Owing to the enhancer action, zidebactam in combination with cefepime (WCK 5222) has been demonstrated to possess potent *in vitro* and *in vivo* activity against highly resistant Gram-negative pathogens including carbapenem-resistant *P. aeruginosa* and *Acinetobacter* (Avery et al., 2018; Karlowsky et al., 2020; Kidd et al., 2020). WCK 5222 is under clinical development for the treatment of Gram-negative infections (NCT02707107 and NCT02674347; www.clinicaltrials.gov) (Mushtaq et al., 2021; Bhagwat et al., 2021; Palwe et al., 2021).

A previous report showed that sulbactam at concentrations as low as 1/4 minimum inhibitory concentration (MIC) in combination with 8 mg/l zidebactam elicited a fast and sustained bactericidal response against an OXA-23-producing *A. baumannii* isolate (Moya et al., 2017). However, the sulbactam/zidebactam combination has not been further explored in a larger number of *A. baumannii* isolates with diverse backgrounds. Considering the limited information on the performance of sulbactam/zidebactam against CRAB and the previously observed good response of this pathogen to the combination of sulbactam with other DBOs, in this work we evaluated the sulbactam/zidebactam combination against CRAB strains, including strains that exhibited resistance against the sulbactam/avibactam combination.

## Material and methods

### Bacterial strains

A total of 43 CRAB clinical strains containing different mechanisms of resistance (OXA-23, OXA-58, IMP-1, NDM-1, IS*Aba1*-OXA-66) including three previously well-characterized strains such as AB5075, AB0057, and AYE (Hujer et al., 2006; Fournier et al., 2006; Jacobs et al., 2014) were used to test sulbactam or sulbactam in combination with zidebactam (**Table 1**). In addition, four genetically constructed deletion variants (AB5075Δ*mreB*, AB5075*ΔadvA*, AB5075*ΔadeB*, AB5075*ΔPBPG*) were used (Manoil Lab, Washington, USA).

**Table 1:** Sulbactam MICs against CRAB strains on cation-adjusted Mueller–Hinton broth with and without zidebactam supplementation.

Strains	Carbapenemase produced	SUL MICs (mg/L)		MIC fold decrease
		CaMHB	CaMHB+ZID4 mg/L	
**AMA 02**	NDM-1	4	1	2
**AMA 07**	NDM-1	32	4	3
**AMA 14**	NDM-1	64	2	4
**AMA 16**	NDM-1	32	2	4
**AMA 26**	NDM-1	8	1	3
**AMA 28**	NDM-1	64	4	4
**AMA 30**	NDM-1	64	4	4
**AMA 33**	NDM-1	64	2	5
**AMA 39**	NDM-1	16	2	3
**AMA 40**	NDM-1	64	4	4
**AMA 47**	NDM-1	16	8	1
**AMA 122**	NDM-1	8	2	2
**AMA 181**	NDM-1	4	2	1
**AMA NO**	NDM-1, OXA-23	128	16	3
**AMA 136**	IMP	4	0.25	4
**AMA 51**	OXA-23	32	1	5
**AMA 113**	OXA-23	16	1	4
**AMA 116**	OXA-23	32	2	4
**AMA 133**	OXA-23	16	2	4
**AMA 147**	OXA-23	16	2	4
**AMA 163**	OXA-58	1	< 0.125	> 3
**AMA 166**	OXA-23	64	8	3
**AMA 190**	OXA-23	4	< 0.125	> 5
**ABUH 606**	OXA-23	32	4	3
**ABUH 628**	OXA-23	32	4	3
**ABUH 696**	OXA-23	16	4	2
**ABUH 698**	OXA-23	32	4	3
**ABUH 702**	OXA-23	8	4	1
**ABUH 712**	OXA-23	16	4	2
**ABUH 719**	OXA-23	32	4	3
**ABUH 752**	OXA-23	16	4	2
**ABUH 754**	OXA-23	16	4	2
**ABUH 758**	OXA-23	32	4	3
**ABUH 785**	OXA-23	32	4	3
**ABUH 796**	OXA-23	16	4	2
**AB0057**	OXA-23	32	2	4
**AB5075**	OXA-23	32	2	4
**AYE**	OXA-23	16	2	3
**ABUH 728**	IS*Aba1*-OXA66	4	4	0
**ABUH 731**	IS*Aba1*-OXA66	8	4	1
**ABUH 746**	IS*Aba1*-OXA66	16	4	2
**ABUH 747**	IS*Aba1*-OXA66	8	4	1
**ABUH 783**	IS*Aba1*-OXA66	4	2	1
**MIC50**	–	16	4	
**MIC90**	–	64	4	

CaMHB, cation-adjusted Mueller–Hinton broth; ZID, zidebactam.

### Antibiotic susceptibility testing

MICs against sulbactam (range 0.12–512 mg/l) and sulbactam plus 4 mg/l zidebactam were determined using the microdilution method according to the Clinical laboratory Standards Institute, CLSI, Standards ((CLSI) CLSI, 2020). Because breakpoints are not available for sulbactam alone; 4 mg/l was applied for this analysis based on the CLSI-susceptible breakpoint of 8/4 mg/l for ampicillin/sulbactam for *Acinetobacter* spp ((CLSI) CLSI, 2020).

## Results

We chose 43 geographically distinct and genetically heterogenous *A. baumannii* isolates that were *bla*_OXA-23_ (n = 22), *bla*_OXA-58_ (n = 1), IS*Aba1*-*bla*_OXA-66_ (n = 5), *bla*_OXA-23 –_ *bla*_NDM-1_ (n = 1), *bla*_IMP-1_ (n = 1), and *bla*_NDM-1_ (n=13) producers and tested them against the sulbactam and sulbactam/zidebactam combination. Nearly 84% of the panel showed a decrease ≥2 dilutions in sulbactam MICs when tested in combination with zidebactam. In addition, the sulbactam/zidebactam combination was able to restore the sulbactam susceptibility in 33/36 (91%) of the sulbactam-resistant isolates (MIC values were equal to or less than 4 mg/l).

All except one of the OXA-23-producing CRAB strains (ABUH 702) showed a decrease ≥2 dilutions in sulbactam MICs when tested in combination with zidebactam (**Table 1**). Finally, all OXA-23 isolates were susceptible to the sulbactam/zidebactam combination. In addition, the sulbactam/zidebactam combination was able to restore sulbactam susceptibility in the three resistant strains harboring IS*Aba1*-*bla*_OXA-66_ (ABUH 731, ABUH 746, and ABUH 747). ABUH 731 and ABUH 747 showed a one-fold decrease dilution in sulbactam MICs, which is expected to be within the inherent error of the methodology (+/- 1 dilution), while a two-fold decrease was seen for ABUH 746 (**Table 1**).

Among *bla*_NDM-1_ producers, a >2-fold decrease in sulbactam MICs was also observed in 11 out of 13 isolates. In AMA NO, which is a strain that harbors both carbapenemases *bla*_OXA-23_ and *bla*_NDM-1_, a fourfold decrease in sulbactam MIC was observed in the sulbactam/zidebactam combination; however, this reduction did not restore susceptibility for sulbactam (**Table 1**).

Considering the carbapenemase produced in the different isolates, the group that showed the weak response to the sulbactam/zidebactam combination was the IS*Aba1*-*bla*_OXA-66_ producers, where one dilution or two dilutions were observed in the resistant strains included.

To further support our observations and using two different medium conditions (CaMHB and Brain Heart infusion (BHI)), an MIC study with a panel of additional 33 CRAB strains was performed. A significant decrease in sulbactam MICs (MIC of ≤4 mg/l) in the presence of zidebactam was observed in 45% and 79% when tested in CaMHB and BHI, respectively (**Table S1**). In BHI broth, the MIC values for the sulbactam/zidebactam combination were lower when compared to those obtained in CaMHB in 24 of the tested strains, while in the rest the values were the same than the ones obtained in CaMHB. Bactericidal synergy was also observed even against *bla*_NDM-1_ + *bla*_OXA-23_ (dual carbapenemase) expressing *Acinetobacter* (**Table S1**) (personal communication).

In addition, four knockout strains (genes involved in peptidoglycan synthesis, cell division proteins, and efflux pumps AB5075Δ*mreB*, AB5075*ΔadvA*, AB5075*ΔadeB*, AB5075*ΔPBPG*) were also tested. We observed an average of fourfold decrease for sulbactam MICs (**Table 2**). All the MIC values for the knockout strains were less than 4 mg/l for the sulbactam/zidebactam combination restoring sulbactam susceptibility. Some of the knockout strains exhibited a fourfold decrease in the MIC in the sulbactam/zidebactam combination (**Table 2**). Remarkably, when we compared with previously published results (Pasteran et al., 2021), we observed loss of avibactam enhancement of sulbactam in this knockout strain (AB5075Δ*mreB*, AB5075*ΔadvA*, AB5075*ΔadeB*, AB5075*ΔPBPG*), suggesting that the sulbactam/zidebactam combination potentially possesses greater efficiency to inhibit these targets or recognize different target genes.

**Table 2:** Sulbactam MICs against *A. baumannii* knockout strains using cation-adjusted Mueller–Hinton broth with and without zidebactam supplementation.

Strains	SUL MICs (mg/L)	
	CaMHB	CaMHB + ZID4mg/L
**AB5075Δ*mreB***	8	1
**AB5075Δ*advA***	32	2
**AB5075Δ*adeB***	32	2
**AB5075Δ*PBPG***	16	1

CaMHB, cation-adjusted Mueller–Hinton broth; ZI, zidebactam.

## Discussion

Since avibactam restored susceptibility to sulbactam in certain CRAB strains and because zidebactam exhibits PBP2 binding and strong synergy when combined with PBP3-binding cefepime or ceftazidime against multidrug-resistant Gram-negative bacteria, we strived to evaluate the synergy of sulbactam and zidebactam against CRAB strains.

The sulbactam/zidebactam combination was able to restore sulbactam susceptibility in 91% of the clinical isolates tested, even in five out of the six strains (AMA113, AMA116, AMA122, AMA133, AMA147) that were resistant to sulbactam/avibactam combination. Zidebactam demonstrated a better enhancement of sulbactam activity compared to avibactam; when sulbactam is combined with avibactam, 69% of the studied *Acinetobacter* spp. isolates were inhibited by 4 mg/l of sulbactam (Pasteran et al., 2021).

In regard to the *bla*_OXA_ producers, a twofold decrease in sulbactam MICs was observed in 79% of the tested isolates. Remarkably, the greatest difference between the combinations of avibactam and zidebactam with sulbactam was observed among NDM producers: zidebactam proved to be effective by decreasing twofold sulbactam MICs in 85% of the NDM-1 producers tested, rendering MIC_50/90_ at 2 and 4 mg/l compared to 32 and 256 mg/l, respectively, achieved with the sulbactam/avibactam combination (Pasteran et al., 2021).

Many studies demonstrated that the zidebactam-mediated potentiation of cefepime activity against Enterobacterales and *P. aeruginosa* isolates is manifested by a significant reduction in cefepime MICs (Barceló et al., 2021; Jean et al., 2022). On the other hand, against *A. baumannii* isolates, such enhancement in the activity is readily perceptible in *in vivo* PK/PD studies that have established that the human-simulated regimen of cefepime/zidebactam combination elicits a potent 2–3-log kill of OXA-carbapenemases expressing *A. baumannii* in neutropenic murine lung/thigh infection even against strains with MIC up to 64 mg/l (Moya et al., 2017; Avery et al., 2018; Almarzoky Abuhussain et al., 2019).

Along similar lines, the potent synergy between sulbactam and zidebactam is also attributed to an “enhancer effect” of zidebactam which results from its high-affinity PBP2 binding in *A. baumannii* and, in combination with PBP3-binding sulbactam, triggers potent bactericidal action (Moya et al., 2017). Zidebactam’s potency in terms of *A. baumannii* PBP2 binding could be judged from the fact that its IC_50_ of 0.01 mg/l is several folds lower than that of imipenem which is a well-documented potent PBP2-binding agent.

Interestingly, unlike other β-lactamase inhibitor combinations, the synergy between sulbactam and zidebactam appears to be independent of β-lactamase expressed by the pathogen and continues to manifest in organisms that produce zidebactam non-inhibitable β-lactamases (such as NDM carbapenemases expressed in *A. baumannii*). The MICs of sulbactam with zidebactam tend to be lower than that of the avibactam combination which points toward the role of potent binding of zidebactam to *A. baumannii* PBP2.

## Conclusion

In the present study, we have tested sulbactam/zidebactam, a combination not largely tested before against *A. baumannii.* The *in vitro* results, with most of isolates displaying MICs under 4 mg/l, compel us to further explore its potential use *in vivo*. The synergy observed with the sulbactam/zidebactam combination exhibited improved results compared with the sulbactam/avibactam combination to restore sulbactam susceptibility against CRAB strains. The sulbactam/zidebactam combination merits further study against CRAB isolates even in those cases where the absence of synergy with other DBOs was observed in microbiological testing.

## Data Availability Statement

The original contributions presented in the study are included in the article/**Supplementary Material**. Further inquiries can be directed to the corresponding author.

## Author Contributions

Conceptualization, FP, AC, MT, RB, and MR; methodology, JC, MB, VF, FP, and SM; formal analysis, FP, SM, GR, RB, and MR; investigation, FP and MR; resources, MT, RB, and MR; writing—original draft preparation, SM, FP, and MR; writing—review and editing, FP, MT, AC, RB, and MR; visualization, FP and MR; supervision, MR; project administration, FP and MR; funding acquisition, FP, MT, RB, and MR. All authors have read and agreed to the published version of the manuscript.

## Funding

Authors’ work cited in this review article was funded by Public Health Service Grants SC3GM125556 (to MR), R01AI100560 (to RB), R01AI063517 (to RB), R01AI072219 (to RB), and R15 AI047115 (to MT) from the National Institutes of Health and VA 1I01BX001974 (to RB) form the Cleveland Department of Veterans Affairs. The content is solely the responsibility of the authors and does not necessarily represent the official views of the National Institutes of Health or the Department of Veterans Affairs. Also, ANLIS: Regular federal budget from Ministry of Health, Argentina. V.F. was supported by Project RAISE, U.S. Department of Education HSI-STEM, award number P031C160152.

## Conflict of Interest

The authors declare that the research was conducted in the absence of any commercial or financial relationships that could be construed as a potential conflict of interest.

## Publisher’s Note

All claims expressed in this article are solely those of the authors and do not necessarily represent those of their affiliated organizations, or those of the publisher, the editors and the reviewers. Any product that may be evaluated in this article, or claim that may be made by its manufacturer, is not guaranteed or endorsed by the publisher.
