# Supplementary material for: Zidebactam restores sulbactam susceptibility against carbapenem-resistant Acinetobacter baumannii isolates
Source: Front Cell Infect Microbiol. 2022 Jul 8;12:918868. doi: 10.3389/fcimb.2022.918868 (PMC9309244; doi:10.3389/fcimb.2022.918868)
Supplement: Supplementary file 1 [file Table_1.docx]

| **Strains** | **β-lactamase Resistance mechanism** | **CaMHB** | **SUL+ZID 4 mg/L** | **BHI** | **SUL+ZID**  **4 mg/L** |
| --- | --- | --- | --- | --- | --- |
| AB NCTC 13303 | OXA-25 | >32 | 4 | 32 | 4 |
| AB NCTC 13304 | OXA-27 | >32 | 2 | 16 | 1 |
| AB NCTC 13301 | OXA-58, OXA-23 | >32 | 8 | 32 | 2 |
| AB ATCC 19606 | ADC, PER | >32 | 0,5 | 16 | 0,5 |
| AB NCTC 13422 | ADC | >32 | 2 | 16 | 1 |
| AB J 145 | OXA-24 | >32 | 1 | 8 | 0,5 |
| AB S 4 | OXA-58, OXA-23 | >32 | 16 | >32 | 4 |
| AB M 1 | OXA-58, OXA-23 | >32 | 8 | >32 | 4 |
| AB M 137 | OXA-58, OXA-23 | >32 | 8 | >32 | 2 |
| AB G 163 | OXA-58, OXA-23 | >32 | 16 | 32 | 4 |
| AB S 11 | OXA-58, OXA-23 | >32 | 2 | >32 | 2 |
| AB G 156 | OXA-58, OXA-23 | >32 | 2 | 16 | 2 |
| AB J 143 | OXA-58, OXA-23 | >32 | 4 | 32 | 4 |
| AB G 169 | OXA-58, OXA-23 | >32 | 1 | 32 | 1 |
| AB M 130 | OXA-58, OXA-23 | >32 | 4 | >32 | 2 |
| AB Q 1466116 | OXA-58, OXA-23 | >32 | 4 | >32 | 2 |
| AB S 225 | OXA-58, OXA-23 | >32 | 8 | >32 | 2 |
| AB Q 1421970 | OXA-58, OXA-23 | >32 | 8 | 32 | 4 |
| AB M 126 | OXA-58, OXA-23 | >32 | 4 | >32 | 1 |
| AB S 6 | OXA-58, OXA-23 | >32 | 16 | >32 | 16 |
| AB Q 1460643 | OXA-58, OXA-23 | >32 | 4 | 32 | 2 |
| AB M 129 | OXA-58, OXA-23 | >32 | 8 | >32 | 2 |
| AB S 9 | OXA-58, OXA-23 | >32 | 2 | 16 | 0,5 |
| AB S 7 | OXA-58, OXA-23 | >32 | 8 | >32 | 8 |
| AB S 8 | OXA-58, OXA-23 | >32 | 8 | 32 | 4 |
| AB G 157 | OXA-58, OXA-23 | >32 | 4 | 32 | 2 |
| AB M 132 | OXA-58, OXA-23 | >32 | 8 | >32 | 2 |
| AB S 15 | OXA-58, OXA-23 | >32 | 8 | >32 | 8 |
| AB S 13 | OXA-58, OXA-23 | >32 | 16 | >32 | 8 |
| AB Y 21 | NDM-1 | >32 | 32 | 32 | 8 |
| AB S 5 | NDM-1, OXA-23 | >32 | 32 | >32 | 8 |
| AB S 224 | NDM-1, OXA-23 | >32 | 32 | >32 | 8 |
| AB S 12 | NDM-1, OXA-23 | >32 | 8 | >32 | 4 |

**Supplementary table 1. Sulbactam MICs *A. baumannii* strains in two different media with and without zidebactam supplementation.**

CaMHB: Cation-Adjusted Mueller–Hinton Broth; BHI: Brain Heart Infusion Broth; SUL: sulbactam; ZID: zidebactam
